# The IgH 3′ regulatory region influences lymphomagenesis in Igλ-Myc mice

**DOI:** 10.18632/oncotarget.3963

**Published:** 2015-04-29

**Authors:** Faten Saad, Alexis Saintamand, Michel Cogné, Yves Denizot

**Affiliations:** ^1^ CNRS UMR 7276, CRIBL, Université de Limoges, Limoges, France

**Keywords:** IgH 3′ regulatory region, Igλ-Myc mice, B-cell lymphoma

## Abstract

The IgH 3′regulatory region (3′RR), encompassing the four transcriptional enhancers hs3a-hs1,2-hs3b-hs4, has a key role on class switch recombination, somatic hypermutation, IgH transcription and B-cell fate. In plasma cells, transcribed IgH and IgL loci often colocalized in transcription factories and an IgL transcription defect might translate into lowered IgH transcription. We explored whether the 3′RR would affect lymphomagenesis in Igλ-Myc transgenic mice prone to lymphoproliferations. Breeding Igλ-Myc transgenics in a background deficient for the 3′RR influences lymphomagenesis toward less mature lymphomas (16% *vs* 54%, *p* = 0.01, Z test for two population proportions). In a 3′RR-deficient background mature tumors less often expressed the CD43 antigen (54% *vs* 0%, *p* = 0.02), a membrane glycoprotein expressed on activated mature B-cells. In contrast, in a 3′RR-deficient background tumors more often expressed the CD5 antigen (32% *vs* 12%, *p* = 0.05) that may serve to control autoimmunity and that is suspected to play a role in leukemic transformation. Lymphoma myc transcript levels, the Ki67 index of proliferation, the clonality, the usage of V(D)J segments, and their somatic hypermutation status were not affected in the 3′RR-deficient background. In conclusion, most probably through its action during the maturation process, the 3′RR can influence lymphomagenesis even when not linked with an oncogene.

## INTRODUCTION

The immunoglobulin heavy chain (IgH) locus undergoes multiple changes along B-cell differentiation, affecting transcription and accessibility to V(D)J recombination, somatic hypermutation (SHM) and class switch recombination (CSR) [1, 2]. Since Ig gene remodelling events require transcription, *cis*-regulatory regions and especially transcriptional enhancers are major locus regulators. The IgH 3′ regulatory region (3′RR) enhancers (hs3a, hs1,2, hs3b and hs4) promote IgH transcription [3], SHM [4] and CSR [5, 6] but not V(D)J recombination [7]. Ongoing recombination and mutation all along B-cell development make the IgH and Ig light (IgL) chain locus hotspots for translocations [8, 9]. Numerous lymphomas are thus marked by proto-oncogene translocation into the IgH locus such as cyclin D1, Bcl-2 and c-myc for mantle cell lymphoma, follicular lymphoma and Burkitt lymphoma, respectively. Convincing demonstration of the key contribution of the IgH and IgL enhancers in mature B-cell lymphomagenesis has been done by transgenic animal models. Thus, c-myc-3′RR and Igλ-Myc transgenics developed Burkitt lymphoma-like proliferation [10-15]. IgH and IgL enhancers may thus be potent activators of IgH/IgL-translocated oncogene transcription, even when breakpoints lie several hundred kb away from the enhancers. Thus, long-range interactions between the two regions of chromatin, through formation of a loop structure constitute an important mechanism of normal and abnormal gene transcription regulation by the 3′RR [2, 16]. Although IgL and IgH chains are encoded by loci located in different chromosome, nuclear positions of Ig alleles are coordinated according to ordered V(D)J recombination [17]. During B-cell activation one IgH allele more often associates with the nuclear periphery [18], while the chromatin context of both alleles appears similar in 4C experiments [19]. Observation of frequent inter-allelic (*trans*-) CSR in mammals also implies inter-allelic proximity favoring recombination and synapsis between alleles [20, 21]. In plasma cells, transcribed IgH and IgL loci often colocalized in transcription factories and an IgL transcription defect might translate into lowered IgH transcription [22]. Until now, the role of the 3′RR in the development of B-cell lymphomas due to a IgL enhancer-mediated oncogene deregulation is unknown. We have explored whether the 3′RR would affect lymphomagenesis in Igλ-Myc transgenic mice prone to lymphoproliferations. The Igλ-Myc transgene induces the B-cell specific overexpression of the human c-myc oncogene leading to the progressive development of B-cell lymphomas [14].

## RESULTS AND DISCUSSION

### Generation of 3′RR-deficient lymphoma mice

**Figure 1:**
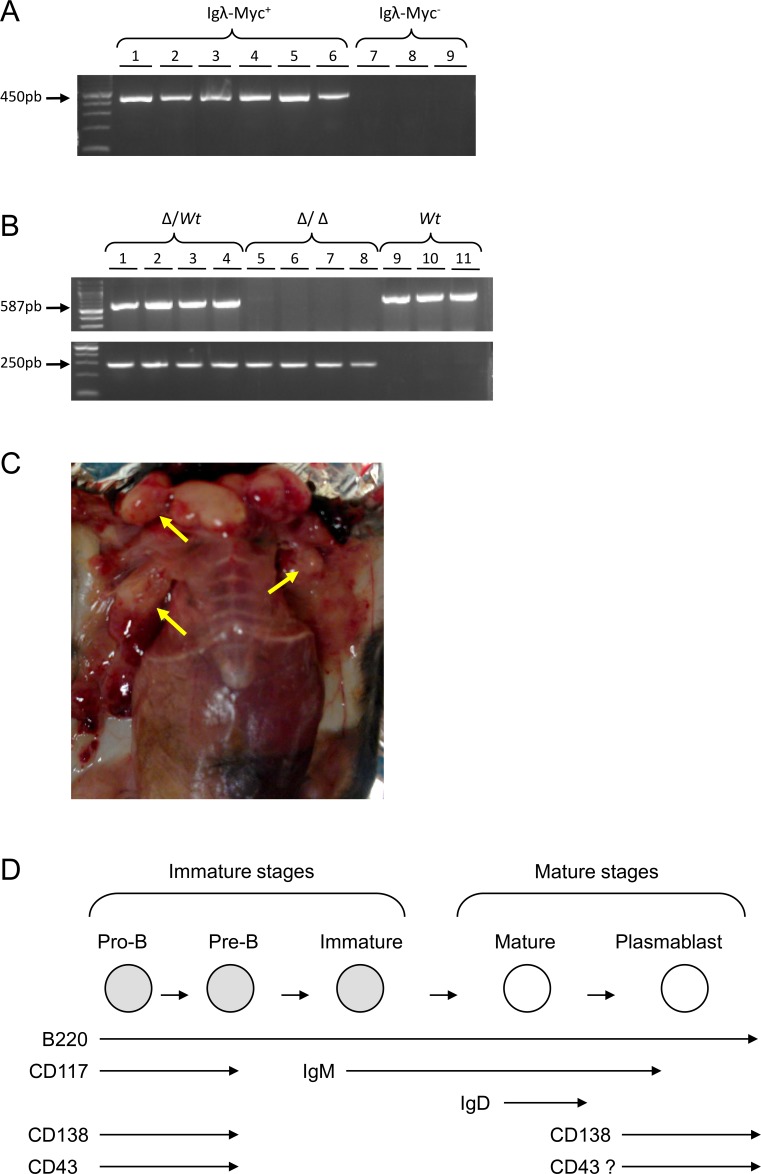
Igλ-Myc/3′RR-deficient lymphoma mice **A.** A typical PCR profile for the detection of the Igλ-Myc transgene. Lines 1-6: six positive mice (amplification of a 450 bp fragment). Lines 7-9: three negative mice. **B.** A typical PCR profile for the detection of the 3′RR-deficient allele (amplification of a 250 bp fragment) and the 3′RR *wt* allele (amplification of a 587 bp fragment). Lines 1-4: four heterozygous 3′RR-deficient mice (∆/*wt*). Lines 5-8: four homozygous 3′RR-deficient mice (∆/∆). Lines 9-11: three *wt* mice. **C.** Tumors in lymphoma mice. Arrows indicate lymphoma presence. **D.** Schematic representation of the B-cell development with the presence of several membrane cell markers. The “?” indicates the potential expression of the CD43 antigen on activated mature B-cells.

3′RR-deficient mice were crossed with Igλ-Myc mice (considered as *wt* in this study) to derive heterozygous 3′RR-deficient/Igλ-Myc and homozygous 3′RR-deficient/Igλ-Myc mice. The presence of the Igλ-Myc transgene (Figure 1A) and of the 3′RR-deleted allele (Figure 1B) was followed by using specific PCR.

### Characteristics of lymphomas in 3′RR-deficient lymphoma mice

**Figure 2:**
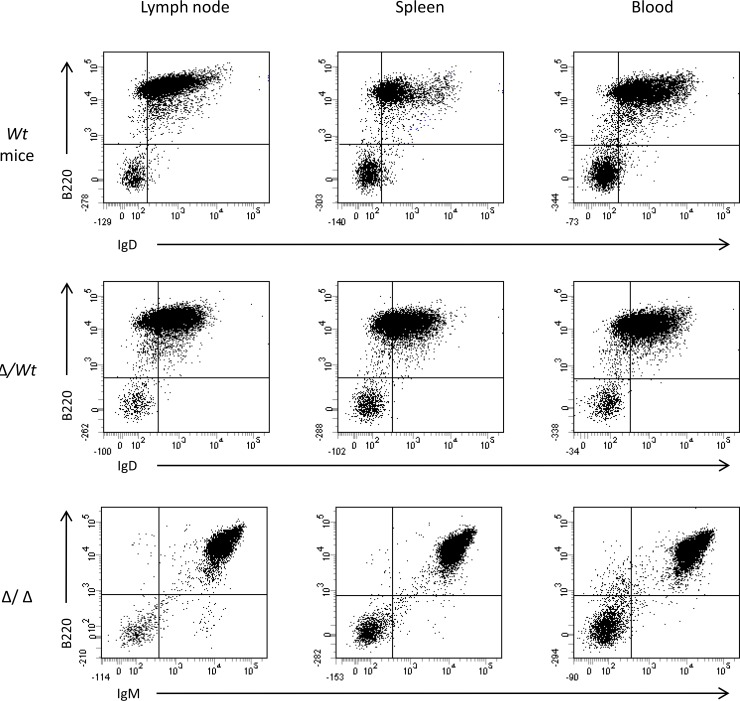
Flow cytometry analysis of lymphoma cells in spleen, lymph node and blood Typical flow cytometry of lymphoma cells from lymph node, spleen and blood of lymphoma mice. Results of a B220/IgD labelling for an Igλ-Myc mice (*wt*), of a B220/IgD labelling for a heterozygous 3′RR-deficient/Igλ-Myc mice (∆/*wt*) and a B220/IgM labelling for an homozygous 3′RR-deficient/Igλ-Myc mice (∆/∆). Three representative profiles from 6 lymphoma mice (2 *wt*, 2 ∆/*wt* and 2 ∆/∆).

After several weeks, mice developed lymphomas with obvious lymph node involvement (Figure 1C). According to the French law animal exhibiting tumors were sacrificed. At necropsy, lymphoma mice had enlarged lymph nodes and spleen. Mice with tumors showed leukemic peripheral blood involvement with circulating lymphoma cells. The numbers of total circulating white blood cells were not different (*p* > 0.05, Mann-Whitney *U*-test) between Igλ-Myc lymphoma mice (136 ± 28 10^3^ cells/μl, *n* = 10), heterozygous 3′RR/Igλ-Myc lymphoma mice (495 ± 194 10^3^ cells/μl, *n* = 17) and homozygous 3′RR/Igλ-Myc lymphoma mice (175 ± 33 10^3^ cells/μl, *n* = 18) but elevated (*p* = 0.02, Mann-Whitney *U*-test) as compared with healthy Igλ-Myc mice (81 ± 5 10^3^ cells/μl, 4 mice) and healthy 3′RR-deficient mice (71 ± 7 10^3^ cells/μl, 4 mice). Flow cytometry analysis of circulating lymphoma cells showed the same labelling pattern than spleen and lymph node lymphoma cells (Figure 2). Means of flow cytometry labelling were also similar (Figure 2) showing that lymphoma cells in lymph nodes, spleen and blood were identical. Lymphoma cells were not associated with liver and gut-associated lymphoid tissues (data not shown). All data reported below were obtained with lymph node lymphomas.

**Figure 3:**
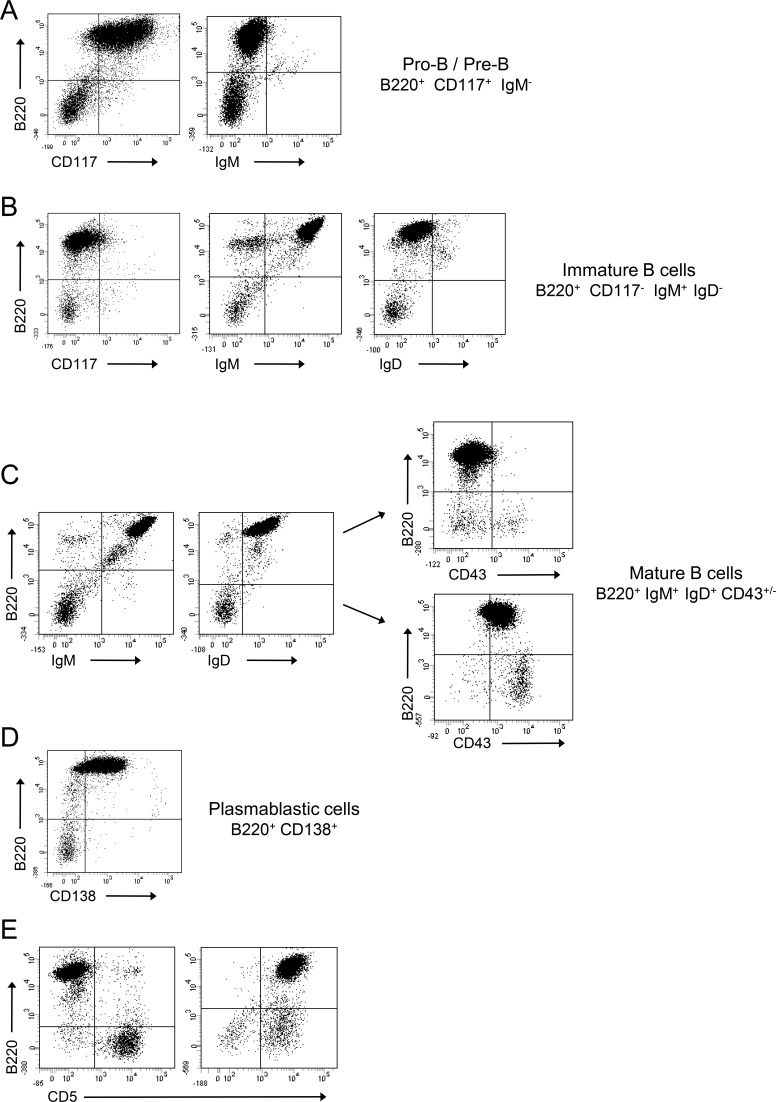
Flow cytometry analysis of Igλ-Myc lymphomas Lymphomas were labelled with various antibodies and analyzed with flow cytometry. Lymphoma analysis revealed four major subtypes. **A.** pro-B/pre-B lymphomas (B220^+^CD117^+^IgM^−^); **B.** immature B-cell lymphoma (B220^+^CD117^−^IgM^+^IgD^−^); **C.** mature B-cell lymphoma (B220^+^IgM^+^IgD^+^ with or without CD43); **D.** plasmablastic B-cell lymphoma (B220^+^CD138^+^). **E**. Some lymphomas were CD5^+^. Representative B220/CD117, B220/IgM, B220/IgD, B220/CD43, B220/CD138 and B220/CD5 labelling were shown. Anti-B220 was conjugated with PC5. Anti-IgM, anti-IgD, anti-CD43 and anti-CD5 were conjugated with FITC. Anti-CD117 and anti-CD138 were conjugated with PE.

**Figure 4:**
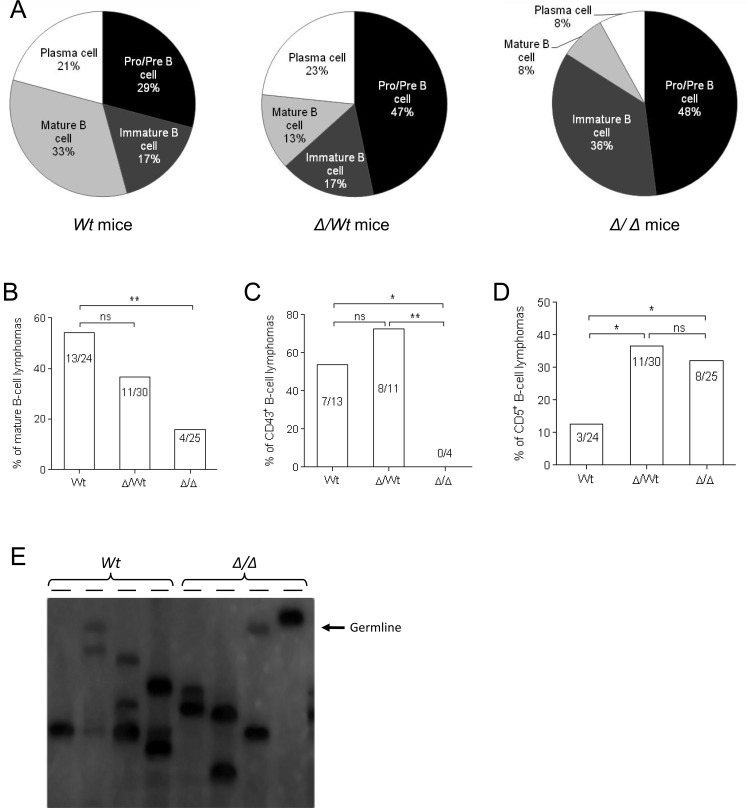
Igλ-Myc lymphomas in a 3′RR-deficient background **A.** Percentage of pro-B/pre-B lymphomas, immature B-cell lymphomas, mature B-cell lymphomas and plasmablastic B-cell lymphomas in Igλ-Myc (*wt*), heterozygous 3′RR-deficient/Igλ-Myc (∆/*wt*) and homozygous 3′RR-deficient/Igλ-Myc mice (∆/∆). **B.** Percentage of total (mature B-cell lymphomas plus plasmablastic B-cell lymphomas) mature B-cell lymphomas in Igλ-Myc (*wt*), heterozygous 3′RR-deficient/Igλ-Myc (∆/*wt*) and homozygous 3′RR-deficient/Igλ-Myc mice (∆/∆). ^*^p < 0.05, Z test for two population proportions. **C.** Percentage of total (mature B-cell lymphomas plus plasmabslastic B-cell lymphomas) CD43^+^ mature B-cell lymphomas in Igλ-Myc (*wt*), heterozygous 3′RR-deficient/Igλ-Myc (∆/*wt*) and homozygous 3′RR-deficient/Igλ-Myc mice (∆/∆). ^*^p < 0.05, ^**^p < 0.005, Z test for two population proportions. **D.** Percentage of total CD5^+^ B-cell lymphomas in Igλ-Myc (*wt*), heterozygous 3′RR-deficient/Igλ-Myc (∆/*wt*) and homozygous 3′RR-deficient/Igλ-Myc mice (∆/∆). ^*^p < 0.05, Z test for two population proportions. **E.** Lymphoma clonality. Southern blot analysis was used to examine lymphoma clonality with a J_H4_ probe. Genomic DNA was prepared and digested with *Eco*RI from lymph node cells of Igλ-Myc mice (*wt*) and homozygous 3′RR-deficient/Igλ-Myc mice (∆/∆). The arrow located the germinal band.

Lymphomas from 79 animals were investigated (24 *wt* mice, 30 heterozygous mice and 25 homozygous mice). During precursor B-cell differentiation, genes encoding H and L chains of an Ig molecule are somatically assembled from germline DNA. This process occurs in the bone marrow prior to antigenic challenge and leads to the successive formation of pro-B, pre-B and immature B-cells. B-cells mature in germinal centers and once activated differentiate into Ig-secreting plasma cells (schematized in Figure 1D). Whether the membrane B220 marker is present all along B-cell maturation, stage specific markers characterise these different stages: expression of the membrane CD117/CD43/CD138 antigens on pro-B/pre-B-cells, expression of membrane IgM on immature B-cells, expression of membrane IgD on mature B-cells, re-expression of membrane CD138 on plasma cells and re-expression of membrane CD43 on activated mature B-cells (schematized in Figure 1D) [23]. Flow cytometry was used to monitor the immunophenotypic profile of lymphomas (typical profiles are reported in Figure 3). A B220^+^ population was characterized in all cases (100%, 79/79) while staining for T lineage (CD4 and CD8) and monocyte/macrophage lineage (CD11b) was negative (data not shown). Percentages of pro-B/pre-B, immature B-cell, mature B-cell and plasmablastic B-cell lymphomas in Igλ-Myc, heterozygous 3′RR/Igλ-Myc and homozygous 3′RR/Igλ-Myc lymphoma mice are reported in Figure 4A. Deletion of the 3′RR significantly (*p* = 0.01, Z test for two population proportions) influenced the lymphoma maturation stage with reduced total (mature B-cell lymphomas plus plasmablastic B-cell lymphomas) mature lymphomas (16%, 4/25) compared to Igλ-Myc *wt* mice (54.1%, 13/24) (Figure 4B). Although reduced (36.6%, 11/30), mature lymphomas in heterozygous 3′RR/Igλ-Myc mice were not significantly different (*p* > 0.05, Z test for two population proportions) compared to Igλ-Myc *wt* mice. CD43 is a sialylated single chain membrane glycoprotein expressed on activated mature B-cells [24]. Deletion of the 3′RR significantly (*p* < 0.05, Z test for two population proportions) reduced the number of CD43^+^ mature B-cell lymphomas. CD43 is thus present on 54% (7/13), 73% (8/11) and 0% (0/4) of total mature B-cell lymphomas of *wt*, heterozygous and homozygous 3′RR-deficient Igλ-Myc mice, respectively (Figure 4C). CD5 is a transmembrane glycoprotein on the surface of some B-cells associated with the B-cell receptor. CD5 might have a major negative influence on antigen receptor driven-B-cell function and may serve to control autoimmunity [25]. The number of CD5^+^ B-cell lymphomas is higher (*p* < 0.05, Z test for two population proportions) in heterozygous 3′RR-deficient Igλ-Myc mice (36%, 11/30) and homozygous 3′RR-deficient Igλ-Myc mice (32%, 8/25) than in *wt* Igλ-Myc mice (3/24, 12%) (Figure 4D).

All along B-lymphocyte development, cell survival is dependent from BCR expression and signalling. IgH transcription is markedly reduced in 3′RR-deficient mice even if BCR expression, signalling and B-cell compartments (except for marginal zone B-cell one) are roughly normal [3]. The lower percentage of total mature B-cell lymphomas in the 3′RR-deficient background might be linked to a lowered maturation speed unmasked by the c-myc-induced lymphoproliferation context. The lower percentage of CD43 activated mature B-cell lymphomas in the 3′RR-deficient background is coherent with this hypothesis. CD5^+^ B-cells have distinct functional properties compared with B-cells lacking CD5. In B-cells, CD5 associates with the BCR and is suspected to maintain anergy in mouse B-cells. CD5 also promotes multiple intracellular signalling pathways in B-lymphocytes [26] and CD5^+^ B-cells are more resistant to apoptose than CD5^−^ B-cells [25]. The elevated CD5^+^ B-cell lymphomas in 3′RR-deficient Igλ-Myc mice might unmask a role of the CD5 antigen in leukemic transformation as previously suggested [26]. The role of the 3′RR deletion on the CD5^+^ B-cell fate deserves now further investigations. Finally, taken altogether these results reinforce a recent study reporting that the class-specific BCR tonic signal modulates lymphomagenesis in a c-myc deregulation transgenic model [13].

### Clonal origin of lymphomas

Southern analysis of V(D)J recombinations showed that all B-cell lymphomas had undergone clonotypic Ig rearrangements. Thus, the use of a J_H4_ probe revealed rearranged bands in addition to the germline band indicating the clonal origin of lymphomas from Igλ-Myc mice with or without the 3′RR (Figure 4E).

### Ki67 expression of lymphomas

The proliferative activity of tumor cells was investigated with the nuclear protein Ki67 found during G_1_, S, G_2_ and M phase of the cell cycle. The percentage of Ki67^+^ cells was high and similar in Igλ-Myc mice (91%, 12 mice), heterozygous 3′RR-deficient/Igλ-Myc mice (87%, 21 mice) and homozygous 3′RR-deficient/Igλ-Myc mice (89%, 21 mice) showing that the intensive lymphoma proliferation was not affected by deletion of the 3′RR. These results are in agreement with the lack of effect of the 3′RR deletion on the growth of normal B-cells [5].

### Mouse and human c-myc transcripts in lymphomas

**Figure 5:**
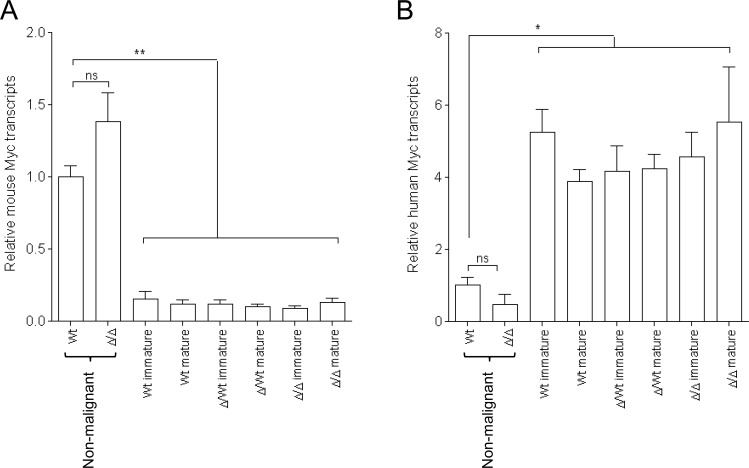
Murine and human c-myc transcripts in Igλ-Myc lymphomas in a 3′RR-deficient background **A.** Murine myc transcripts **B.** Human myc transcript in immature and mature B-cell lymphomas in Igλ-Myc (*wt*), heterozygous 3′RR-deficient/Igλ-Myc (∆/*wt*) and homozygous 3′RR-deficient/Igλ-Myc mice (∆/∆). Five immature and five mature B-cell lymphomas for *wt* mice. Eight immature and eleven mature B-cell lymphomas for ∆/*wt* mice. Eleven immature and five mature B-cell lymphomas for ∆/∆ mice. Pre-malignant splenic B-cells of four *wt* and four ∆/∆ mice were used as controls. *p < 0.01 and **p < 0.001 (Mann-Whitney *U*-test).

Igλ-Myc mice carried an Igλ-Myc transgene containing a translocated MYC gene from the human Burkitt lymphoma cell line IARC-BL60 under the transcriptional control of the IgL λ chain regulatory sequences [14]. Thus the dual analysis of both human transgenic myc transcripts and endogenous mouse myc transcripts can be investigated in Igλ-Myc mice. We analyzed mouse splenic B-cells in 8 week old homozygous 3′RR-deficient/Igλ-Myc mice and Igλ-Myc mice before any manifestation of disease. Mouse and human myc transcripts levels in splenic B-cells were not affected by the 3′RR-deficient background (Figure 5A and 5B). Similarly, the shift between mouse and human myc transcript levels in B-cell lymphomas of Igλ-Myc mice was not affected. For these experiments purified splenic B-cells of 8-weeks old mice prior any manifestation of disease were used as controls. This was done because lymphomas arose from axillary, parotid, and submandibular lymph nodes (Figure 1C) and that in steady state conditions it is impossible to obtain sufficient amounts of purified lymph node B-cells from 8-weeks old mice. Finally, human myc transcript levels were similar in immature and mature B-cell lymphomas (Figure 5B). Taken together these results suggest that the tumoral process is not affected by the 3′RR-deficient background, reinforcing the hypothesis that the lowered lymphoma maturity in 3′RR-deficient/Igλ-Myc mice is related to a difference on cell maturity of the cell of origin of the B-cell lymphoma.

### Analysis of Ig VH mutations in lymphomas

We then analysed the mutation status of V_H_ rearranged genes in B-cell lymphomas. This study was made possible because of their clonal nature (Figure 4E). The 150 bp located downstream the J_H4_ exon were investigated as an hot spot of somatic hypermutation [4]. All lymphomas revealed unmutated sequences and thus featured a pre-germinal centre origin (results from four *wt* lymphomas, three heterozygous 3′RR lymphomas, three homozygous 3′RR lymphomas, ten sequences for each lymphoma) (data not shown). The lack of somatic hypermutations in lymphomas from Igλ-Myc mice is in agreement with their absence in lymphomas from iMyc^Eμ^ mice [27] and reinforced the hypothesis that most mice lymphomas arise from naïve B-cells. It is interesting to note that most human B-cell lymphomas are mutated suggesting that the cell of origin of human and mouse lymphomas differs suggesting multifactorial route to tumor development and that correlations between human lymphomas and mice models should be made cautiously.

### Analysis of V(D)J recombination in lymphomas

**Figure 6:**
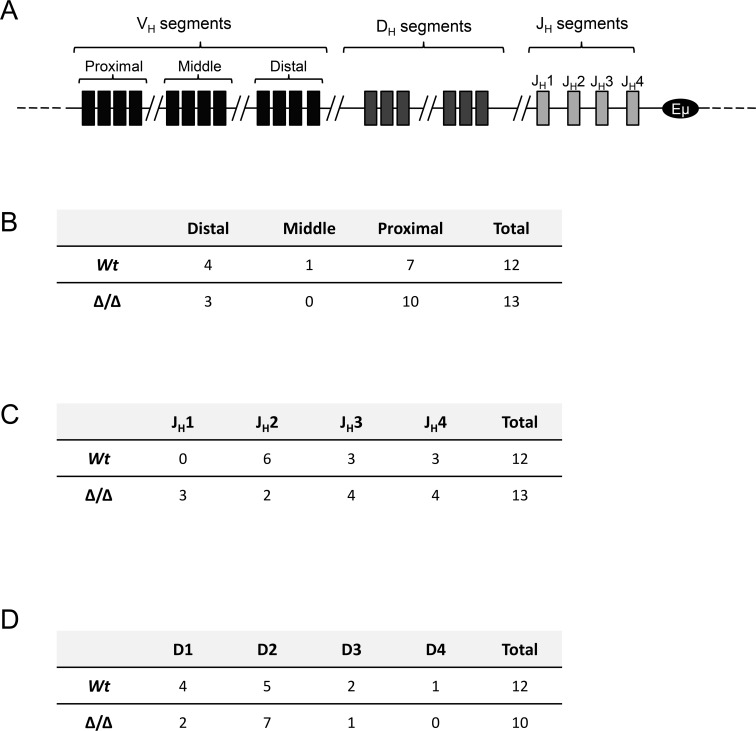
V(D)J usage in Igλ-Myc lymphomas in a 3′RR-deficient background **A.** Schematic representation of segments of variability (V), diversity (D) and junction (J) in the IgH mouse locus. **B.** Analysis of distal, middle or proximal V genes in twelve lymphomas from Igλ-Myc mice and thirteen lymphomas from homozygous 3′RR-deficient/Igλ-Myc mice. **C.** Analysis of J segments in twelve lymphomas from Igλ-Myc mice and thirteen lymphomas from 3′RR-deficient/Igλ-Myc mice. **D.** Analysis of D segments in twelve lymphomas from Igλ-Myc mice and thirteen lymphomas from 3′RR-deficient/Igλ-Myc mice. Three D segments from lymphomas from 3′RR-deficient/Igλ-Myc mice cannot be determined after amplification, cloning and sequencing of the V-D-J segment due to high mutations/insertions/deletions located at the V-D and D-J junctions.

The mouse IgH locus contains about 200 variable (V_H_) genes subdivided into domain-organized gene families, including the distal V_H_ (the largest family) and the proximal V_H_ family. V_H_ genes are followed by a dozen of diversity (D) segments and four junction (J) segments. We then analysed if 3′RR deficiency affected the type of V, D and J used to express the B-cell receptor at the membrane of B-cell lymphomas. This study was made possible because of their clonal nature. As shown in Figure 6, lack of the 3′RR did not affect the use of either V, D and J segments. This study confirms that the 3′RR is not implicated in V(D)J recombination confirming previous studies using non lymphoma B-cells [7, 28].

## CONCLUDING REMARKS

Results of the present study indicate that the IgH 3′RR influences lymphomagenesis in Igλ-Myc mice. The 3′RR did not directly act on the lymphomagenetic process but on the maturity of the cell of origin of the lymphoma. The 3′RR is a major lymphoma oncogene deregulator [8, 10-13]. Targeted inhibition of the 3′RR could provides a therapeutic strategy for the treatment of a wide range of mature B-cell lymphomas, specifically those with oncogene translocation into the IgH locus [29, 30].

## MATERIALS AND METHODS

### Generation of transgenic mice

3′RR-deficient mice [5] were crossed with Igλ-Myc mice [14] to derive heterozygous 3′RR-deficient/Igλ-Myc mice and homozygous 3′RR-deficient/Igλ-Myc mice. Mice exhibiting obvious tumors or presenting signs of illness were immediately sacrificed. Our research has been approved by our local ethics committee review board (Comité Régional d'Ethique sur l'Expérimentation Animale du Limousin, Limoges, France) and carried according the European guidelines for animal experimentation.

### PCR

PCR experiments for detection of the *wt* 3′RR allele were carried out with specific forward 5′-CCAAAAATGGCCAGGCCTAGG-3′ and reverse 5′-GACCCTGTCCTATGGCT GAC-3′ primers [23]. PCR experiments for detection of the deficient 3′RR allele were carried out with specific forward 5′-TCCCTGGACAATCTGCACAT-3′ and reverse 5′-GACCCTGTCCTATGGCTGAC-3′ primers [30]. Amplification products were analysed on a 1.2% agarose gel. Expected sizes of amplified products were 250 bp and 587 bp for mutated and *wt* alleles, respectively. PCR experiments for detection of the Igλ-Myc transgene were carried out with specific forward 5′-GCTCGTCTCAGAGAAGCTGG-3′ and reverse 5′-ATCTCTCCAGATCTGCTATCTC-3′ primers. Amplification products were analysed on a 1.2% agarose gel. Expected size of amplified products was 450 pb.

### Flow cytometry analysis

Single-cell suspensions from lymph node tumors and spleen were labelled with PC5 anti-B220, PE anti-CD117, FITC anti-CD43, FITC anti-IgM, FITC anti-IgD, FITC anti-CD5, PE anti-CD138, PE anti-CD4, PC5 anti-CD8 and FITC anti-CD11b antibodies (Southern Biotechnologies) and analyzed on a Fortessa LSR2 (Beckman Coulter) [31, 32]. For intracellular labelling experiments, cells were fixed and permeabilized with the Intraprep^TM^ permeabilization reagent (Beckman Coulter) according to the manufacturer's recommendations prior to incubation with FITC anti-Ki67 (Becton Dickinson) or irrelevant antibodies (Cell signalling Technology, Inc).

### Clonality assay

Genomic DNA prepared from lymph node lymphomas was digested with *Eco*RI and analysed by Southern blot with a ^32^P-labeled J_H_ probe [10].

### mRNA expression

Total RNA was extracted from lymph node lymphomas. RNA was reverse-transcribed into cDNA by addition of reverse transcriptase to 2 μg total RNA in a final volume of 20 μl. Real time PCR was performed in duplicate by using TaqMan assay reagents and analysed on an ABI Prism 7000 system (Applied Biosystems Foster City, CA) [32]. Product reference: mouse c-*myc*, Mm00487803-m1; human c-myc, Hs00153408-m1. Mouse actin (Mn00607939-s1) was used for normalization of gene expression levels (Applied Biosystems). In another set of experiments, RNA was extracted from splenic B-cells (purified by CD43 magnetic cell sorting) of 8 week old Igλ-Myc/3′RR-deficient mice and Igλ-Myc mice (*i.e.,* before any manifestation of disease).

### Sequence analysis of expressed V(D)J rearrangements

Genomic DNA extracted from tumors was amplified by PCR. Forward primers: V_H_J558 5′-GCGAAGCTTARGCCTGGG RCTTCAGTGAAG-3′, V_H_Q52 5′-GCGAAGCTTCTCACA GAGCCTGTCCATCAC-3′, V_H_7183 5′ - CGGTACCAAGAASAMCCTGTWCCTGCAAATGASC - 3′ and backward primer: J_H4_ 5′-AGGCTCTGAGATCCCTAGACAG-3′. The PCR products were cloned into the Zero Blunt^®^ Topo^®^ PCR cloning (Invitrogen). Plasmids were isolated using the NucleoSpin kit (Macherey-Nagel Eurl) and sequenced using an automated laser fluorescent ANA ABI-PRISM sequencer (Perkin-Elmer).
